# Carotene hydroxylase DcCYP97A3 affects carotenoid metabolic flow and taproot color by influencing the conversion of α-carotene to lutein in carrot

**DOI:** 10.1093/hr/uhaf054

**Published:** 2025-02-18

**Authors:** Hui-Ru Wang, Rong-Rong Zhang, Ya-Hui Wang, Jian-Hua Zhou, Miao Sun, Li-Xiang Wang, Yu-Qing Zhang, Yi Liang, Xiao-Jie Li, Zhi-Sheng Xu, Jing Ma, Hui Liu, Jian-Ping Tao, Ai-Sheng Xiong

**Affiliations:** State Key Laboratory of Crop Genetics and Germplasm Enhancement and Utilization, Ministry of Agriculture and Rural Affairs Key Laboratory of Biology and Germplasm Enhancement of Horticultural Crops in East China, College of Horticulture, Nanjing Agricultural University, 1 Weigang, Nanjing 210095, China; State Key Laboratory of Crop Genetics and Germplasm Enhancement and Utilization, Ministry of Agriculture and Rural Affairs Key Laboratory of Biology and Germplasm Enhancement of Horticultural Crops in East China, College of Horticulture, Nanjing Agricultural University, 1 Weigang, Nanjing 210095, China; State Key Laboratory of Crop Genetics and Germplasm Enhancement and Utilization, Ministry of Agriculture and Rural Affairs Key Laboratory of Biology and Germplasm Enhancement of Horticultural Crops in East China, College of Horticulture, Nanjing Agricultural University, 1 Weigang, Nanjing 210095, China; Institute of Agricultural Science and Technology of Zhengzhou, 6 Changjiang Road, Zhengzhou 450005, China; State Key Laboratory of Crop Genetics and Germplasm Enhancement and Utilization, Ministry of Agriculture and Rural Affairs Key Laboratory of Biology and Germplasm Enhancement of Horticultural Crops in East China, College of Horticulture, Nanjing Agricultural University, 1 Weigang, Nanjing 210095, China; College of Marine and Biological Engineering, Yancheng Teachers University, 2 Xiwang Road, Yancheng 224002, China; State Key Laboratory of Crop Genetics and Germplasm Enhancement and Utilization, Ministry of Agriculture and Rural Affairs Key Laboratory of Biology and Germplasm Enhancement of Horticultural Crops in East China, College of Horticulture, Nanjing Agricultural University, 1 Weigang, Nanjing 210095, China; State Key Laboratory of Crop Genetics and Germplasm Enhancement and Utilization, Ministry of Agriculture and Rural Affairs Key Laboratory of Biology and Germplasm Enhancement of Horticultural Crops in East China, College of Horticulture, Nanjing Agricultural University, 1 Weigang, Nanjing 210095, China; Vegetable Research Center, Beijing Academy of Agriculture and Forestry Sciences, 11 Shuguang Garden Road, Beijing 100097, China; Vegetable Research Center, Beijing Academy of Agriculture and Forestry Sciences, 11 Shuguang Garden Road, Beijing 100097, China; State Key Laboratory of Crop Genetics and Germplasm Enhancement and Utilization, Ministry of Agriculture and Rural Affairs Key Laboratory of Biology and Germplasm Enhancement of Horticultural Crops in East China, College of Horticulture, Nanjing Agricultural University, 1 Weigang, Nanjing 210095, China; State Key Laboratory of Crop Genetics and Germplasm Enhancement and Utilization, Ministry of Agriculture and Rural Affairs Key Laboratory of Biology and Germplasm Enhancement of Horticultural Crops in East China, College of Horticulture, Nanjing Agricultural University, 1 Weigang, Nanjing 210095, China; State Key Laboratory of Crop Genetics and Germplasm Enhancement and Utilization, Ministry of Agriculture and Rural Affairs Key Laboratory of Biology and Germplasm Enhancement of Horticultural Crops in East China, College of Horticulture, Nanjing Agricultural University, 1 Weigang, Nanjing 210095, China; State Key Laboratory of Crop Genetics and Germplasm Enhancement and Utilization, Ministry of Agriculture and Rural Affairs Key Laboratory of Biology and Germplasm Enhancement of Horticultural Crops in East China, College of Horticulture, Nanjing Agricultural University, 1 Weigang, Nanjing 210095, China; State Key Laboratory of Crop Genetics and Germplasm Enhancement and Utilization, Ministry of Agriculture and Rural Affairs Key Laboratory of Biology and Germplasm Enhancement of Horticultural Crops in East China, College of Horticulture, Nanjing Agricultural University, 1 Weigang, Nanjing 210095, China

## Abstract

The color diversity of non-purple carrot taproots is mainly affected by carotenoid species and content. Carrot cytochrome P450 carotene β-ring hydroxylase (DcCYP97A3) may influence carotenoid accumulation in carrots; however, the roles of *DcCYP97A3* in carrot remain unclear. Compared to the orange carrot ‘Kurodagosun, KRD’, the yellow carrot ‘Yellowstone, YST’ had greater relative transcript levels of *DcCYP97A3*. DcCYP97A3 was shown to catalyze the β-ring hydroxylation of α-carotene to create zeaxanthin when it was expressed in *Escherichia coli* accumulating α- and β-carotene. Expression of the *DcCYP97A3* of ‘YST’ in DcCYP97A3 functionally deficient orange carrot ‘KRD’ resulted in yellow taproots, decreased α-carotene and β-carotene content, decreased α-/β-carotene ratio, and increased lutein content. In carrots overexpressing the *DcCYP97A3* gene, the transcript levels of *DcLCYE* and *DcLCYB1* were significantly upregulated and downregulated, respectively. Gene editing of *DcCYP97A3* in ‘YST’ resulted in *DcCYP97A3* knockout mutants with significantly reduced levels of lutein and β-carotene and significantly upregulated transcript levels of *DcCHXB2* and *DcCCD4*. These findings advance our knowledge of the molecular mechanisms behind carrot carotenoid metabolism.

## Introduction

In nature, carotenoids are abundant and can be produced by any photosynthetic organism, such as algae, cyanobacteria, and higher plants [1]. More than 800 natural carotenoids have been discovered and reported. Carotenoids have many conjugated double bonds, are primarily polymerized from eight isoprene units, and exhibit a spectrum of colors in visible light, including red, orange, and yellow, depending on their quantity [2, 3].

The two primary classes of carotenoids are xanthophylls and carotenes; xanthophylls are oxygenated derivatives of carotenes. For instance, lutein, β-cryptoxanthin, and zeaxanthin are xanthophylls, whereas lycopene, α-carotene, and β-carotene are carotenes. Carotenoids are precursors to several important phytohormones, including strigolactones (SLs) and abscisic acid, and are necessary for photosynthesis and photoprotection [1]. Moreover, carotenoids are good for human health because they act as a precursor to the manufacture of vitamin A, which can effectively boost immunity and prevent night blindness [4]. Most animals, including humans, are unable to produce carotenoids, and they must be obtained through food [5].

The carrot (*Daucus carota* L.) is a variant of the wild carrot and an important vegetable crop belonging to the Apiaceaefamily [6–8]. The first domesticated carrots were purple or yellow, and after a long period of domestication, colorful carrot varieties such as orange and red gradually appeared. Except for purple carrots, whose taproot color is governed by the anthocyanin content [9–11], other carrot taproot colors are regulated by the quantity and diversity of carotenoids. For instance, carrots are high in β- and α-carotene when they are orange, high in lutein when they are yellow, and high in lycopene when they are red [12–15].

Carotene hydroxylases in plants can be divided into two main classes, namely heme-containing cytochrome P450 carotene hydroxylase (CYP97A, CYP97B, and CYP97C) and non-heme di-iron carotene hydroxylase (BCH). They catalyze the hydroxylation of the β- and ε-rings on the C3 of different carotenoids. Currently, studies have been conducted to investigate the catalytic function of CYP97A and its effect on carotenoid accumulation in some plants. Within *Arabidopsis*, AtCYP97A3 possesses the capacity to hydroxylate the β-ring of α- and β-carotene, and it also exhibits a mild catalytic activity toward the ε-ring of α-carotene [14, 16]. In *Escherichia coli*, OsCYP97A4 from rice can hydroxylate the β-ring of both α- and β-carotene [17]. When *OsCYP97A4* was knocked out in rice, the amount of lutein in the leaves decreased and the amount of α-carotene increased [18]. In tomato, downregulation of *CYP97A29* gene expression also caused an increase in α-carotene content [19]. No enzymatic activity of CitCYP97A from citrus was detected in *E. coli*, and it was hypothesized that it might be caused by the lack of some cofactors in *E. coli* for CitCYP97A to exert its activity [20].

*DcCYP97A3* may be connected to the amount of carotenoids in carrots, according to previous studies. Overexpression of *AtCYP97A3* in orange carrot roots that had lost DcCYP97A3 function resulted in a decrease in the amount of α-carotene and total carotenoids in the carrot roots, as well as in the α-/β-carotene ratio [21]. The *Y* and *Y_2_* gene models proposed by previous studies showed that when both genes were homozygous and recessive, the taproot of carrot could accumulate β-carotene and lutein at the same time and show orange color [22–25]. In a previous study, a QTL locus on chromosome 7 was identified as significantly affecting the accumulation of β-carotene, α-carotene, phytoene, and total carotenoids in carrot roots in the context of recessive genes for both the *Y* and *Y_2_* genes, and this locus contained the *DcCYP97A3* gene [26]. In this study, the function of *DcCYP97A3* was verified by bioinformatic analysis, prokaryotic expression system, gene overexpression, and CRISPR/Cas9 gene editing techniques. It is important to confirm the role of carrot *DcCYP97A3* to better understand the mechanism underlying the accumulation of carotenoids in carrots.

## Results

### Cloning and analysis of *DcCYP97A3*

Based on the carrot genome database, DNA sequences near the 1074th nucleotide on the exon of the *DcCYP97A3* in orange carrot ‘Kurodagosun’ (‘KRD’) and yellow carrot ‘Yellowstone’ (‘YST’) were obtained by polymerase chain reaction (PCR) and sequencing, respectively. Compared with ‘YST’, there was 8-nucleotide insertion on the exon of *DcCYP97A3* in ‘KRD’ (Fig. S1a). The open reading frame (ORF) sequence of *DcCYP97A3*, which codes for 616 amino acids, was cloned from ‘YST’ (**PQ870845;**  Fig. S2). The conserved domain of DcCYP97A3 amino acid sequence was predicted by InterPro, and the results showed that there was a conserved CYP97 domain at positions 139–572, belonging to the cytochrome P450 family. DcCYP97A3 clustered with KAK1391004.1 from *Heracleum sosnowskyi* of the Apiaceae family, according to the phylogenetic analysis (Fig. S1b). CYP97A proteins from 12 different plants share 76.28% amino acid sequence identity with DcCYP97A3, according to the multiple comparison analysis (Fig. S1c). This suggests that these proteins sequences are relatively conserved.

### Subcellular localization of DcCYP97A3

*Agrobacterium* was used to temporarily transform the pSPYE-*DcCYP97A3* construct into tobacco in order to observe the subcellular localization of DcCYP97A3. Laser scanning confocal microscopy was used to observe the lower epidermis of tobacco leaves, and the spontaneous red fluorescence of chloroplasts overlapped with the green fluorescence of EGFP, indicating that DcCYP97A3 was located in chloroplasts (Fig. S3b).

### Expression analysis of *DcCYP97A3* in orange carrot ‘KRD’ and yellow carrot ‘YST’

**Figure 1:**
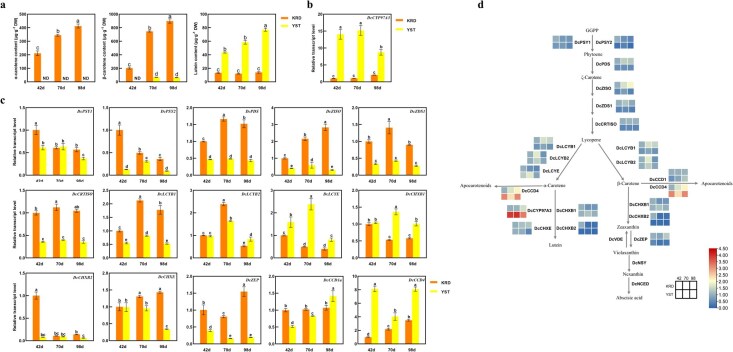
**The expression pattern of carotenoid metabolism genes in ‘KRD’ and ‘YST’ during development**. (a) The contents of α-carotene, β-carotene, and lutein in taproots of ‘KRD’ and ‘YST’. 42, 70, and 98 d represent days after sowing. The relative transcript levels of *DcCYP97A3* (b) and other carotenoid metabolism genes (c) in taproots of ‘KRD’ and ‘YST’. Different letters in graphs a–c denote significant differences [one-way analysis of variance (ANOVA)] at the *P* < 0.05 level, and error bars represent standard deviation across the three biological replicates. (d) Heat map of relative expression levels of carotenoid metabolism pathway genes in two carrot cultivars. Data of the heat map were based on the log2(copy number) values after homogenization.

Ultra performance liquid chromatography (UPLC) was used to measure the amounts of carotenoids in orange carrot ‘KRD’ and yellow carrot ‘YST’ at different growth stages. Whereas ‘YST’ accumulated mostly lutein and β-carotene, ‘KRD’ accumulated primarily α- and β-carotene and a little quantity of lutein (Fig. 1a). Expression levels of carotenoid metabolism genes of the two carrot cultivars ‘YST’ and ‘KRD’ were examined (Fig. 1b–d). According to quantitative real-time PCR (RT-qPCR), at all three developmental stages, ‘YST’ had a higher *DcCYP97A3* transcript level than ‘KRD’. Other carotenoid metabolism genes including *DcPSY1*, *DcPSY2*, *DcPDS*, *DcZISO*, *DcZDS1*, *DcCRTISO*, *DcLCYB1*, *DcCHXB2*, *DcCHXE*, and *DcZEP* had higher transcript levels in ‘KRD’ compared to ‘YST’, but *DcLCYE*, *DcCHXB1*, and *DcCCD4* had lower transcript levels in ‘KRD’ compared to ‘YST’.

### Enzymatic activity analysis of DcCYP97A3 in *E. coli*

**Figure 2:**
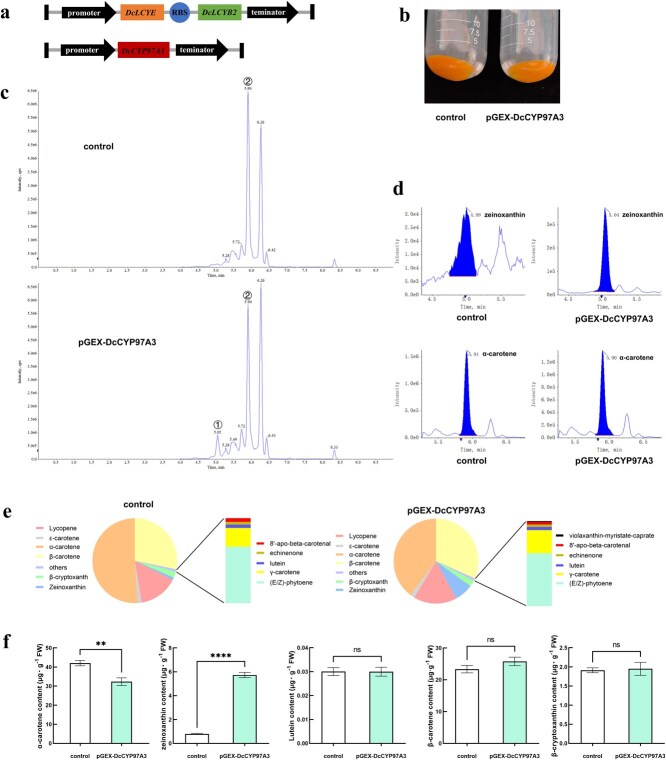
**Prokaryotic expression of carrot DcCYP97A3 protein in *E. coli*.** (a) The schematic diagram of the pET-*DcLCYE* + *DcLCYB2* and pGEX-*DcCYP97A3* constructs. (b) The appearance of *E. coli*. ‘control’ represents *E. coli* with cotransformed of pACCRT-EIB and pET-*DcLCYE* + *DcLCYB2* plasmids; ‘pGEX-DcCYP97A3’ represents *E. coli* cotransformed the pACCRT-EIB, pET-*DcLCYE* + *DcLCYB2*, and pGEX-*DcCYP97A3* plasmids. (c) The TIC (Total Ion Chromatogram) of carotenoid accumulation in *E. coli*: (1) zeinoxanthin peaks at this retention time; (2) α-carotene peaks at this retention time. (d) The integral correction diagram of zeinoxanthin and α-carotene in *E. coli.* (e) Carotenoid distribution in *E. coli*. (f) The contents of α-carotene, zeinoxanthin, lutein, β-carotene, and β-cryptoxanthin in *E. coli*. In graph f, asterisks denote significant differences from the control (^**^P < 0.01, ^****^P < 0.0001; Student’s *t-*test), and error bars represent standard deviation across the three biological replicates.

*DcLCYE* and *DcLCYB2* were ligated by a piece of ribosome binding site (RBS) sequence to be expressed under the same promoter to obtain the pET-*DcLCYE* + *DcLCYB2* construct; *DcCYP97A3* was constructed onto pGEX-5X-1 to obtain pGEX-*DcCYP97A3* construct (Fig. 2a). pACCRT-EIB and pET-*DcLCYE* + *DcLCYB2* were cotransformed into *E. coli* to produce α-carotene and β-carotene, which were used as control. The *E. coli* cotransformed with pGEX-*DcCYP97A3* and the above two plasmids were used as an experimental group to investigate the enzymatic activity of DcCYP97A3. The color of *E. coli* in both control and experimental groups was orange, and there was no obvious difference in visual observation (Fig. 2b). The carotenoid content of *E. coli* in the two groups was measured, and the α-carotene content of the experimental group was significantly decreased than control, which was ~10 μg·g^−1^ FW less than the control group. Zeinoxanthin, which is the product of β-ring hydroxylation of α-carotene, was found in the experimental group in quantities approximately seven times greater than in the control group. The amounts of lutein, β-cryptoxanthin, and β-carotene were less in the two groups, but there was no significant change in their contents. Furthermore, zeaxanthin was not found in the *E. coli* from the experimental or control groups (Fig. 2c–f; Supplemental Tables 1 and 2).

### *DcCYP97A3* overexpression in orange carrot ‘KRD’

**Figure 3:**
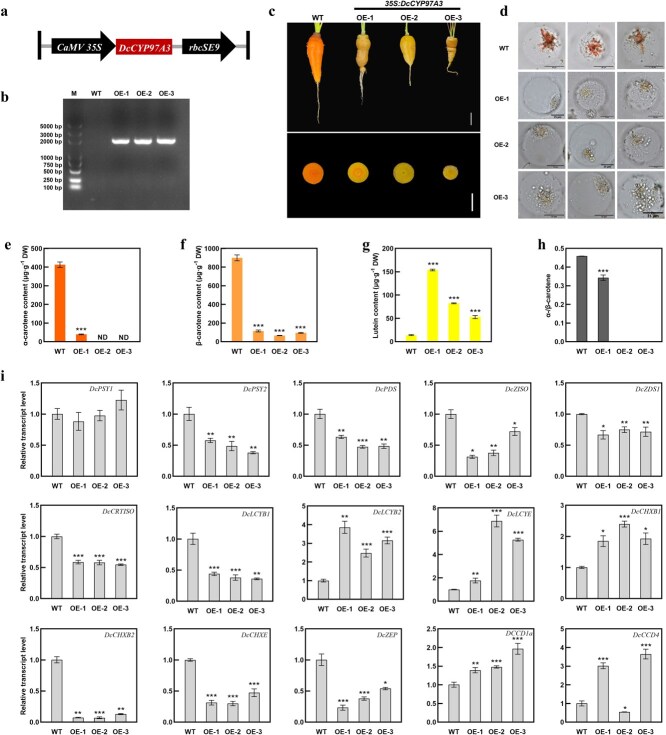
**Impacts of *DcCYP97A3*-overexpression on the metabolism of carotenoid in orange carrot ‘KRD’.** (a) The diagram of *35S:DcCYP97A3* construct. (b) Identification of *DcCYP97A3-*overexpression carrots. M, marker. (c) Control and *DcCYP97A3-*overexpression carrots. The white lines stand for 2 cm. (d) The chromoplasts in control and *DcCYP97A3-*overexpression carrots. The black lines represent 25 μm. α-Carotene (e), β-carotene (f), and lutein (g) content in taproots of *DcCYP97A3-*overexpression carrots. ND represents not detected. (h) The ratio of α-/β-carotene in taproots of *DcCYP97A3-*overexpression carrot lines. (i) Relative transcript levels of carotenoid metabolic genes in taproots of control and *DcCYP97A3-*overexpression carrots. In graphs e–i, asterisks denote significant differences from the control (^*^P < 0.05, ^**^P < 0.01, ^***^P < 0.001; Student’s *t* test), and error bars represent standard deviation across the three biological replicates.

To examine the impact of *DcCYP97A3* on carrot carotenoid accumulation, the *35S:DcCYP97A3* recombinant construct was created by cloning the ORF sequence of *DcCYP97A3* from ‘YST’ into the pCAMBIA1301 vector (Fig. 3a). Subsequently, three *DcCYP97A3*-overexpression lines were generated when orange carrot ‘KRD’ was overexpressed with *DcCYP97A3* (Fig. 3b).

In terms of phenotype, the color of *DcCYP97A3*-overexpression carrots was yellow, while the control was orange (Fig. 3c). The protoplasts of carrot taproots were extracted, and then the morphology of the chromoplasts was observed. It was found that the chromoplasts of control carrot were orange crystals, whereas those of *DcCYP97A3* transgenic carrots were yellow or gray globules (Fig. 3d).

The carotenoid content in *DcCYP97A3*-overexpression carrots was further determined. Compared with control carrots, transgenic carrots contained significantly less α-carotene and β-carotene and significantly more lutein (Fig. 3e–g) and α-carotene accumulation was not detected in the taproots of OE-2 or OE-3. The amount of α-carotene in OE-1 was 39.34 μg·g^−1^ DW, which was 9.53% of that in control carrot (Fig. 3e). The amounts of β-carotene in OE-1, OE-2, and OE-3 were 12.76%, 7.38%, and 10.58% of those in control carrots, respectively (Fig. 3f). The amount of lutein in OE-1, OE-2, and OE-3 were 11.50, 5.83, and 3.76 times of control carrot (Fig. 3g). Additionally, *DcCYP97A3*-overexpression carrots had a lower α-/β-carotene ratio (Fig. 3h).

To further understand how overexpression of *DcCYP97A3* affects carotenoid metabolism, the relative transcript levels of carotenoid metabolic genes were measured (Fig. 3i). The three transgenic carrot lines hosting *DcCYP97A3* gene showed no significant change in *DcPSY1* transcript levels compared to the control carrot. *DcPSY2*, *DcPDS*, *DcZISO*, *DcZDS1*, *DcCRTISO*, *DcLCYB1*, *DcCHXB2*, *DcCHXE*, and *DcZEP* transcript levels were downregulated slightly or significantly. Among them, the relative transcript levels of *DcCHXB2* were most significantly downregulated. The relative transcript levels of *DcLCYB2*, *DcLCYE*, *DcCHXB1*, and *DcCCD1a* showed different degrees of upregulation. The relative transcript levels of *DcLCYB2* in OE-1, OE-2, and OE-3 were 3.86, 2.48, and 3.16 times of those in control carrot, respectively. The relative transcript level of *DcLCYE* in OE-2 and OE-3 was 6.88 and 5.28 times that of control carrot, respectively. The relative transcript level of *DcCCD4* was significantly upregulated in OE-1 and OE-3, which was 3.02 and 3.65 times of control carrot, respectively. In OE-2, the relative transcript level of *DcCCD4* was downregulated, accounting for 54.5% of the control carrot.

### *Pro_KRD_:DcCYP97A3* transgenic carrot

The promoter sequences of *DcCYP97A3* were cloned from ‘KRD’ (**PQ870843;**  Supplemental Table 7) and ‘YST’ (**PQ870844;**  Supplemental Table 7), and the sequencing results showed that the lengths were 2147 and 1979 bp, respectively. Compared to ‘KRD’, the promoter sequence of ‘YST’ has two long fragment deletions, in addition to some SNPs and small fragment differences (Fig. S4).

**Figure 4:**
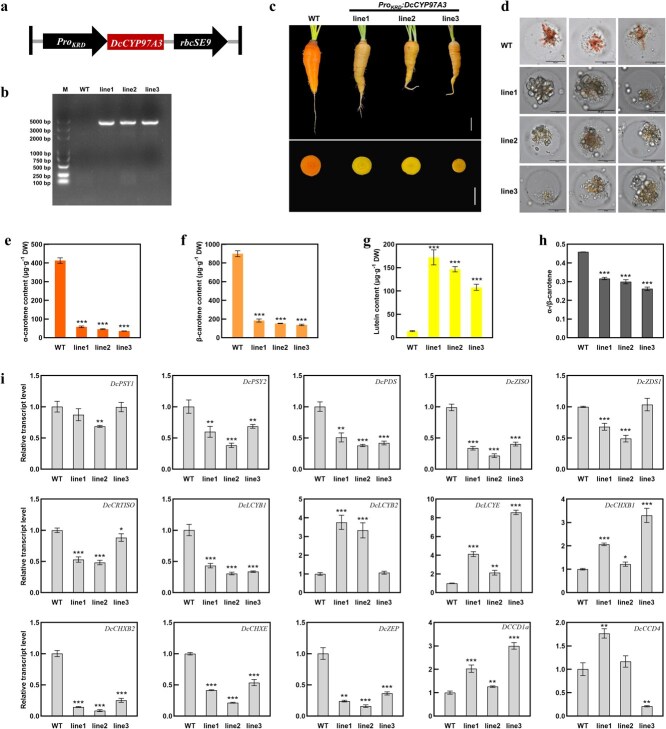
***Pro***_***KRD***_ ***:DcCYP97A3* transgenic carrot.** (a) The diagram of *Pro_KRD_:DcCYP97A3* construct. (b) Identification of *Pro_KRD_:DcCYP97A3* lines. M, marker. (c) Control and *Pro_KRD_:DcCYP97A3* transgenic ‘KRD’ carrots. The white lines stand for 2 cm. (d) The chromoplasts in control and *Pro_KRD_:DcCYP97A3* transgenic lines. The black lines represent 25 μm. α-Carotene (e), β-carotene (f), and lutein (g) content in taproots of *Pro_KRD_:DcCYP97A3* transgenic lines. (h) The ratio of α-/β-carotene in taproots of *Pro_KRD_:DcCYP97A3* transgenic lines. (i) Relative transcript levels of carotenoid metabolic genes in taproots of control and *Pro_KRD_:DcCYP97A3* transgenic lines. In graphs e–i, asterisks denote significant differences from the control (^*^P < 0.05, ^**^P < 0.01, ^***^P < 0.001; Student’s *t-*test), and error bars represent standard deviation across the three biological replicates.

The promoter region of *DcCYP97A3* was cloned from ‘KRD’, and ORF of *DcCYP97A3* was cloned from ‘YST’ to prepare *Pro_KRD_:DcCYP97A3* construct (Fig. 4a). Then, the construct was transformed into ‘KRD’, and three transgenic lines hosting *Pro_KRD_:DcCYP97A3* (Line 1, Line 2, and Line 3) were identified by PCR amplification result (Fig. 4b).

The taproot color of control carrot (KRD) is orange, and the taproot color of the *Pro_KRD_:DcCYP97A3* transgenic carrot is yellow (Fig. 4c). The chromoplasts in the taproots of control carrot are orange crystals, while the chromoplasts in the taproots of *Pro_KRD_:DcCYP97A3* transgenic carrot are yellow or gray globules, and the transgenic carrots hosting *Pro_KRD_:DcCYP97A3* contained some amyloplasts (Fig. 4d).

Similarly, the carotenoid content in *Pro_KRD_:DcCYP97A3* carrots was determined (Fig. 4e–g). The α-carotene content of Line 1, Line 2, and Line 3 were 14.12%, 11.13%, and 8.68% of control, respectively, indicating a significant decrease in the amount of α-carotene in *Pro_KRD_:DcCYP97A3* carrots as compared to control (Fig. 4e). The β-carotene content of *ProKRD:DcCYP97A3* carrots was also significantly lower than control, which were 20.47%, 17.04%, and 15.24% of control, respectively (Fig. 4f). The lutein content of *Pro_KRD_:DcCYP97A3* carrots was significantly increased, which were 12.19, 10.39, and 7.65 times of control respectively (Fig. 4g). Additionally, *ProKRD:DcCYP97A3* carrots had a considerably lower α-/β-carotene ratio than the control group; the ratios in Lines 1, 2, and 3 decreased to 0.32, 0.30, and 0.26, respectively (Fig. 4h).

The relative transcript levels of carotenoid metabolism genes in *Pro_KRD_:DcCYP97A3* carrots were further investigated (Fig. 4i). The relative transcript level of *DcPSY1* was slightly downregulated in Line 2. The relative transcript levels of *DcPSY2*, *DcPDS*, *DcZISO*, *DcZDS1*, *DcCRTISO*, *DcLCYB1*, *DcCHXB2*, *DcCHXE*, and *DcZEP* were slightly or significantly downregulated, and the relative transcript level of *DcCHXB2* was the most downregulated. The transcription levels of *DcLCYB2*, *DcLCYE*, *DcCHXB1*, and *DcCCD1a* were upregulated to varying degrees in *Pro_KRD_:DcCYP97A3* carrots, and the relative transcript levels of *DcLCYB2* in Line 1 and Line 2 were 3.76 and 3.33 times of control, respectively. The relative transcript levels of *DcLCYE* in Line 1 and Line 3 were 4.12 and 8.58 times of control, respectively. The relative transcript level of *DcCHXB1* in Line 1 and Line 3 is 2.07 and 3.30 times of control, respectively. The relative transcript level of *DcCCD4* showed different trends in different transgenic lines. It was upregulated in Line 1 and downregulated in Line 3, while there was no significant change in Line 2.

### Gene editing of *DcCYP97A3* in yellow carrot ‘YST’

**Figure 5:**
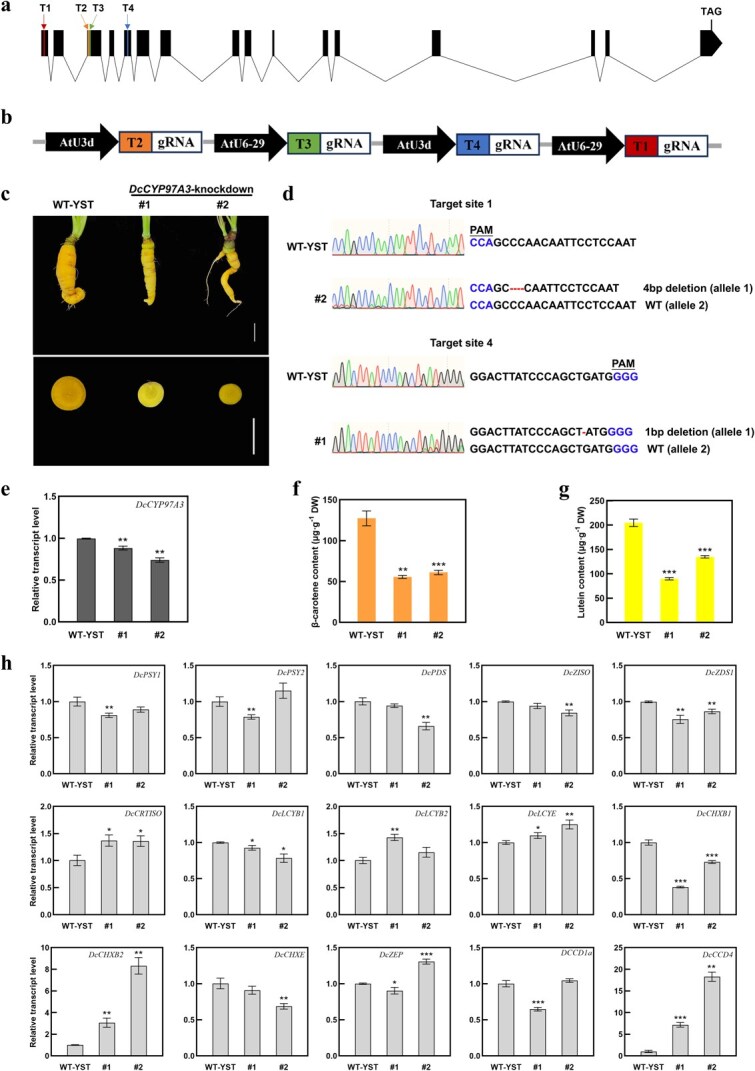
**Knocking out *DcCYP97A3* in ‘YST’.** (a) Schematic representation of the location of *DcCYP97A3* gene editing targets. The *DcCYP97A3* gene’s exons and introns are denoted by black boxes and black lines, respectively. T1, T2, T3, and T4 were the target sites. (b) Assembly structure diagram of four sgRNA expression cassettes. (c) Control and *DcCYP97A3*-knockdown carrots. The white lines stand for 2 cm. (d) Sequences of successfully edited sites in *DcCYP97A3*-knockdown carrots. (e) Expression levels of *DcCYP97A3* in control and *DcCYP97A3*-knockdown carrots. β-Carotene (f) and lutein (g) content of control and *DcCYP97A3*-knockdown carrots. (h) Relative transcript levels of carotenoid metabolic genes in control and *DcCYP97A3*-knockdown carrots. In graphs e–h, asterisks denote significant differences from the control (^*^P < 0.05, ^**^P < 0.01, ^***^P < 0.001; Student’s *t*-test), and error bars represent standard deviation across the three replicates.

CRISPR/Cas9 technology was used to knock out the *DcCYP97A3* gene in ‘YST’ in order to better understand its function (Fig. 5). Based on the genomic reference sequence of *DcCYP97A3*, we designed four target sites, of which T1 was located in Exon 1, T2 and T3 in Exon 3, and T4 in Exon 5 (Fig. 5a). The four target sites and gRNAs were driven by two types of promoters, *AtU3d* and *AtU6–29*, respectively (Fig. 5b). Two heterozygous mutants, #1 and #2, were obtained based on the results of mutation detection at the target site (Fig. 5d).

The *DcCYP97A3* knockdown mutants had a lighter yellow color than the control, which was comparatively dark yellow (Fig. 5c). The relative transcript levels of *DcCYP97A3* in the *DcCYP97A3* knockdown mutants were lower than the control (Fig. 5e). Carotenoids were measured in the taproots of the *DcCYP97A3* knockdown mutants, and significant decreases in β-carotene and lutein content were found (Fig. 5f and g). In knockdown mutants #1 and #2, the amount of β-carotene was 43.66% and 48.00% of the control carrot, respectively (Fig. 5f), and the amount of lutein was 43.86% and 65.89% of the control carrot, respectively (Fig. 5g).

Relative transcript levels of some carotenoid metabolism genes were changed in the knockdown mutants compared to controls (Fig. 5h). The transcript levels of *DcCHXB2* and *DcCCD4* were significantly upregulated in #1 and #2, where the transcript levels of *DcCHXB2* were 3.07 and 8.33 times of control, and the transcript levels of *DcCCD4* were 7.17 and 18.27 times of control, respectively. In addition, the transcript levels of *DcLCYE* were slightly upregulated and those of *DcLCYB1* and *DcCHXB1* were slightly downregulated in knockdown mutants.

## Discussion

Diversity in carotenoid composition and content can lead to different colors among some plant varieties [27–29]. The cytochrome P450 carotene β-ring hydroxylase encoded by *CYP97A* is involved in the conversion of carotenoids into xanthophylls. The catalytic function of CYP97A in different species may be different. In *Arabidopsis*, AtCYP97A3 mainly catalyzed the β-ring hydroxylation of α-carotene and β-carotene, and it also has a weak hydroxylation effect on the ε-ring on α-carotene [16]. Expression of rice *OsCYP97A4* gene in *E. coli* showed that OsCYP97A4 could catalyze β-ring hydroxylation of α-carotene and β-carotene, but could not catalyze ε-ring hydroxylation of α-carotene [17]. It has been found that in some orange carrot varieties, eight nucleotides were inserted into the coding sequence of the *CYP97A3* gene, leading to premature termination of translation and encoding only 382 amino acids, resulting in a loss of gene function and affecting the content of α-carotene and total carotenoids [21]. This study identified the same insertion mutation in *DcCYP97A3* that causes loss of gene function in orange carrots (KRD), but no insertion mutation in yellow carrots (YST). Further bioinformatics analysis revealed that DcCYP97A3 contains one CYP97 structural domain and the amino acid sequence is more conserved with higher homology with other species. In plants, carotenoids are synthesized primarily on chromoplasts. It has been demonstrated that in rice the β-ring hydroxylase CYP97A4 is localized in chloroplasts [18], DcCYP97A3 is also localized in chloroplasts, which indicates that DcCYP97A3 has normal hydroxylase activity.

In taproots of ‘KRD’ and ‘YST’, carotenoid accumulation and carotenoid metabolism gene expression levels at different developmental stages were further compared. The carotenoid contents of the two cultivars varied, with lutein accumulating primarily in ‘YST’ and β- and α-carotene accumulating primarily in ‘KRD’. The transcript level of *DcCYP97A3* in ‘YST’ was significantly higher than that in ‘KRD’. In ‘KRD’ and ‘YST’, there were differences in gene structure and transcript levels of *DcCYP97A3*, which may account for the difference in carotenoid accumulation in the two different colored carrots.

To learn more about the catalytic function of the DcCYP97A3 enzyme, *DcCYP97A3* was expressed in *E. coli* that accumulated α- and β-carotene [15, 30]. The results showed that DcCYP97A3 could catalyze the generation of zeinoxanthin from α-carotene, indicating that DcCYP97A3 has a hydroxylating effect on the β-ring of α-carotene. Due to the limitation of experimental conditions, we could not measure the content of α-cryptoxanthin, the product of ε-ring hydroxylation of α-carotene. However, there was no significant change in the amount of lutein, the hydroxylation product of the β- and ε-ring of α-carotene, so we hypothesized that DcCYP97A3 might not be able to catalyze the hydroxylation of the e-ring of α-carotene. Furthermore, there were no significant changes in the amounts of β-carotene and its hydroxylation product, β-cryptoxanthin, as well as no zeaxanthin detected. These findings suggest the possibility that DcCYP97A3 has no hydroxylation activity on the β-ring of β-carotene. There are some limitations of the *E. coli* prokaryotic expression assay additionally. For example, no hydroxylation activity appears in *E. coli* when citrus CYP97A was expressed, which was hypothesized to be probably caused by the lack of some cofactors in *E. coli* for CitCYP97A to exert its activity [20]. Therefore, whether DcCYP97A3 has secondary catalytic activity also needs to be further explored.

To verify the function of *DcCYP97A3* in carotenoid acumination, we further overexpressed *DcCYP97A3*, cloned from yellow carrot ‘YST’, in orange carrot ‘KRD’. Taproots with overexpression of *DcCYP97A3* had a decreased amount of α- and β-carotene and increased amounts of lutein. *DcLCYE* controls the transformation of carrot lycopene into α-carotene [15]. Carrot taproots’ β-carotene concentration increased when *DcLCYB1* was overexpressed in orange carrots, whereas *DcLCYB1* silencing had the opposite effect [31]. The transcript levels of *DcLCYE* were significantly upregulated and those of *DcLCYB1* were downregulated in *DcCYP97A3-*overexpression carrots, suggesting that there was an increase in lycopene flow to α-carotene and a decrease in flow to β-carotene. Increased lycopene flow to the α-branch, coupled with overexpression of *DcCYP97A3*, ultimately leads to increased levels of lutein, a downstream product of α-carotene, and decreased levels of α-carotene. The decrease in lycopene flow to the β-branch leads to a decrease in the production and content of β-carotene.

PSY is an important rate-limiting enzyme in the carotenoid synthesis pathway. The accumulation of carotenoids in the orange carrot callus can be inhibited by *DcPSY2* knockout, suggesting that *DcPSY2* is essential for carrot carotenoids synthesis [32]. Here, the transcription levels of *DcPSY2* and some genes related to early carotenoid synthesis (*DcPDS*, *DcZISO*, *DcZDS1*, *DcCRTISO*) were downregulated in *DcCYP97A3*-overexpression carrots as compared to control carrot. The downregulation of *DcPSY2* transcription level explains the reduction in total carotenoid content, and this similar feedback regulation has also been reported in other studies, where overexpression of *AtCYP97A3* in orange carrot root with loss of DcCYP97A3 function decreased PSY protein level, leading to a reduction in total carotenoids [21]. Overexpression of the *AcBCH* gene from kiwifruit in tomato also resulted in a downregulation of *PSY* transcript levels and a decrease in total carotenoids in tomato fruit [33].

A previous study has found that both *DcCHXB1* and *DcCHXB2* have catalytic carotene β-ring hydroxylation activity in carrots, and it was hypothesized that *DcCHXB1* and *DcCHXB2* are biased toward hydroxylation of the β-ring of β-carotene and α-carotene, respectively [34]. A possible explanation for the decrease in carrots’ α- and β-carotene quantity is the significantly increased transcript level of *DcCHXB1* in *DcCYP97A3*-overexpression vegetables carrots. The significant downregulation of the transcript level of *DcCHXB2* may be caused by the competitive effect of *DcCYP97A3* on the substrate α-carotene. DcCCD1 can cleave δ-carotene and β-carotene to generate α- and β-ionone in *E. coli* [35]. *DcCYP97A3-*overexpression carrots showed an increase in *DcCCD1a* transcript level, which could be linked to a decrease in β-carotene. Additionally, the transcript levels of *DcZEP* were decreased among the three transgenic lines, and it is hypothesized that overexpression of *DcCYP97A3* may also affect the xanthophyll cycle [36].

The *DcCYP97A3* gene has a small fragment insertion leading to protein loss of function in orange carrot ‘KRD’. The ORF of *DcCYP97A3* from the yellow carrot ‘YST’ with normal function was ligated in promoter of ‘KRD’, *Pro_KRD_*, and then transformed into ‘KRD’. The phenotype of *Pro_KRD_:DcCYP97A3* carrots was similar to that of *DcCYP97A3*-overexpression carrots. This suggests that *Pro_KRD_* in ‘KRD’ has promoter activity and can drive *DcCYP97A3* expression, and the main factor affecting the accumulation of carotenoids in ‘KRD’ is the coding region of the *DcCYP97A3* gene rather than its promoter. However, compared to ‘KRD’, ‘YST’ had a higher *DcCYP97A3* transcript level, and the promoter sequences of ‘KRD’ and ‘YST’ differed to a certain extent. Differences in *cis*-elements on promoters cause differences in gene transcription levels and thus differences in pigment accumulation in plant tissues. For example, deletion of the *cis*-elements on the promoter of cotton *GhRPRS1* led to a decrease in its transcriptional activity, thus changing the petal color from red to yellow or white [37]. Mutations in the *DcMYB7* or *DcMYB113* promoter led to a significant reduction in its transcriptional level, resulting in a nonpurple coloration of carrot taproots [9, 10]. Therefore, whether the difference in the promoter sequences of the *DcCYP97A3* between the two carrot varieties would result in different transcript levels of the *DcCYP97A3* needs to be further verified. In addition, transcription factors regulate gene transcription by binding to *cis*-elements of the promoter. *cis*-elements such as multiple light-responsive elements and MYB-binding sites were also present on the *DcCYP97A3* promoter region, and thus whether the differences in *DcCYP97A3* transcript levels between the two carrot varieties were caused by certain upstream transcriptional regulators also needs to be further explored.

*DcCYP97A3* knockdown mutants had considerably less lutein and β-carotene than control. Similarly, *Arabidopsis lut5-1*, a T-DNA insertion mutant of the *CYP97A3* gene, showed decreased levels of lutein and β-carotene [38]. We found that a small amount of lutein was still accumulated in orange carrot ‘KRD’ with a loss of function of DcCYP97A3, suggesting that there are other genes that may overlap with the function of DcCYP97A3. A previous study have found that *DcCHXB2* may be biased toward having a hydroxylating effect on α-carotene [34]. It is possible that the roles of DcCHXB2 and DcCYP97A3 may overlap because the expression of *DcCHXB2* was significantly downregulated in *DcCYP97A3* transgenic carrots, while it was significantly upregulated in the *DcCYP97A3* knockdown mutants. In the *DcCYP97A3* knockdown mutant, *DcCHXB2* may compensate for part of the *DcCYP97A3* loss of function by upregulating its transcription. A previous study has found that *DcCCD4* can cleave α-carotene and β-carotene [39]. In *DcCYP97A3* knockdown mutant, the expression of *DcCCD4* was significantly upregulated, which may be used to increase carotenoid cleavage to maintain homeostasis of carotenoid metabolism. Our study showed that α-carotene is a catalytic substrate of DcCYP97A3, but no α-carotene was detected in the *DcCYP97A3* knockdown mutant, which may be related to the upregulation of the expression of the *DcCHXB2* and *DcCCD4* on the one hand, and on the other hand, there is also a *DcCYP97A3* allele that has normal function in the knockdown mutants.

Because our results showed that *DcCYP97A3* can significantly affect the carotenoid content and the color of carrot taproots, we further discussed its relationship with the genetic control of carotenoid content. A recent study has identified the *Y_2_*, *Or*, and *REC1* as key loci determining the accumulation of high levels of α- and β-carotenoids, which correlate with the orange phenotype of cultivated carrots [14]. The candidate gene at the *Y_2_* locus, *EX1-like* (DCAR_730022), is homologous to *EXECUTER1*, which mediates the response to singlet oxygen in chloroplast [40]. The candidate gene at *REC1* locus, *REC1-like* (DCAR_206039), is homologous to *Arabidopsis REDUCED CHLOROPLAST COVERAGE 1* (*REC1*), and a *REC1* homologous gene (*RCP2*) in *Mimulus* directly affects the carotenoid content [41]. The relationship of the *Y_2_* locus and the *REC1* locus to the *DcCYP97A3* gene needs further exploration.

## Conclusion

In summary, this study found that DcCYP97A3 can catalyze the hydroxylation of the β-ring of α-carotene in *E. coli*. The function of *DcCYP97A3* was validated using transgene and knockout to influence the metabolic flux of carotene and alter the amounts of α-carotene, β-carotene, and lutein. Overexpression of *DcCYP97A3* gene in orange carrot ‘KRD’ could decrease the contents of α-carotene and β-carotene and increase the contents of lutein. *DcCYP97A3* from ‘YST’ was ligated to *Pro_KRD_* and transformed into ‘KRD’, which was found to have a similar phenotype to carrots overexpressing *DcCYP97A3*, indicating that *Pro_KRD_* has normal promoter activity. In the *DcCYP97A3* knockdown mutant, the contents of lutein and β-carotene were significantly decreased, but no α-carotene was detected. The results can provide theoretical basis for analyzing the carotenoid accumulation mechanism of carrot.

## Materials and methods

### Plant materials

The plant materials were ‘Kurodagosun, KRD’ (an orange carrot cultivar) and ‘Yellowstone, YST’ (a yellow carrot cultivar). Carrots were grown in the smart greenhouse with growth conditions set as 12 h light/12 h dark at 28°C. Carrot taproots were harvested at 42, 70, and 98 days after sowing and frozen in liquid nitrogen and stored at −80°C for subsequent analysis.

### Genomic DNA and total RNA extraction and cDNA preparation

The genomic DNA, total RNA, and First-strand cDNA were obtained as previously described [15].

### Gene cloning and sequence analysis

The gDNA of ‘KRD’ and ‘YST’ were used as templates, and specific primers were designed for PCR amplification of DNA sequence fragments of *DcCYP97A3* gene, and the correct bands were sent to sequencing to detect the presence or absence of insertion mutations in *DcCYP97A3* gene. The cDNA of *DcCYP97A3* was cloned from ‘YST’ using specific primers based on the reference sequence of *DcCYP97A3* (DCAR_023843) in the carrot genome database [23]. The sequences of all the above primers are shown in Supplemental Table 3. The conserved domain of the amino acid sequence of carrot DcCYP97A3 was analyzed by InterPro (https://www.ebi.ac.uk/interpro/search/sequence/). The Multiple amino acid comparison analysis was performed using DNAMAN 9.0. The phylogenetic tree was constructed using MAGE11.0.

### Subcellular localization analysis

The ORF sequence of *DcCYP97A3* without stop codon from ‘YST’ was amplified and used to construct the pSPYE-*DcCYP97A3* construct. Then the construct was transformed into *Agrobacterium tumefaciens* GV3101 and transiently transformed into tobacco (*Nicotiana benthamiana*) and observed for fluorescence as described in previous study [42].

### Quantitative real-time PCR analysis

The primers used for carotenoid metabolism gene expression analysis were designed using the Primer Premier 6.0 software or referenced from previous study (Supplemental Table 4) [43]. The RT-qPCR was performed as previous study described [15]. *DcActin* was used as the reference gene [44], and the 2^-△△Ct^ method was used to calculate relative transcript levels for carotenoid metabolism genes [45].

### Carotenoid extraction and UPLC analysis from carrot

Total carotenoids from carrot taproots were extracted with acetone and measured by UPLC. The extraction and measurement methods were carried out with reference to previous study [39].

### Prokaryotic expression of *DcCYP97A3* in *E. coli*

Three plasmids were used in this experiment, and their specific information was as follows:

1. The pACCRT-EIB Plasmid, which enables the production and accumulation of lycopene when expressed in *E. coli*, was kindly donated by Prof. Norihiko Misawa of Ishikawa Prefectural University, Japan. [46, 47].
2. pET-*DcLCYE* + *DcLCYB2*: The *DcLCYE* gene from red carrot ‘BHJS’ was constructed into pET30a construct in previous study [15]. On the basis of this plasmid, the *DcLCYB2* gene of ‘KRD’ was constructed into the construct, and an RBS sequence was added in the middle of *DcLCYE* gene and *DcLCYB2* gene, so that the two genes were expressed under the action of the same promoter. RBS is a ribosome binding site that can initiate protein translation.
3. pGEX-*DcCYP97A3*: The ORF sequence of *DcCYP97A3* from ‘YST’ was cloned and constructed into the pGEX-5X-1 vector.

To produce α-carotene and β-carotene as substrates, the pACCRT-EIB and the pET-*DcLCYE* + *DcLCYB2* were cotransformed into *E. coli* BL21 (DE3). To investigate the enzyme activity of DcCYP97A3, the pGEX-*DcCYP97A3* and the above two constructs were transformed into *E. coli*. Positive colonies were cultured in LB liquid medium containing the appropriate antibiotics at 37°C to OD600 = 0.6 and induced with 1 mM isopropyl-β-d-thiogalactopyranoside (IPTG) for 24 h at 28°C under dark conditions. *E. coli* was collected for further carotenoid extraction and measurement.

### Carotenoid extraction and LC–MS/MS analysis from *E. Coli*

Carotenoids in *E. coli* were extracted with *n*-hexane/acetone/ethanol (1:1:1, v/v/v) buffer containing 0.01% 2,6-di-tert-butyl-4-methylphenol (BHT). Carotenoids from *E. coli* were analyzed by liquid chromatography tandem mass spectrometry (LC–MS/MS) [48]. The extraction and measurement methods were referred to previous study [15].

### Generation of transgenic and gene-edited carrots

The ORF of *DcCYP97A3* from ‘YST’ was cloned for the construction of the *35S:DcCYP97A3* construct. The promoter of *DcCYP97A3* from ‘KRD’ and the ORF of *DcCYP97A3* from ‘YST’ were cloned for the construction of the *Pro_KRD_:DcCYP97A3* construct. The online website (http://skl.scau.edu.cn/targetdesign/) was used to design the target sites for gene editing, and four target sites were chosen for sgRNA expression cassette construction (Supplemental Table 5). These plasmids were transformed into ‘KRD’ or ‘YST’ by *Agrobacterium*-mediated transformation methods described in a previous study [49]. PCR and sequencing were used to validate the transgenic resistance-positive plants, and the primers used are shown in Supplemental Table 3.

### Chromoplast isolation and observation

The protoplasts of the taproots of carrots were extracted as described in a previous study [50]. The morphology of protoplasts was observed with Axio Imager M2 microscope (Zeiss, Germany).

## Compliance with Ethics Requirement

This article does not contain any studies with human or animal subjects.

## Supplementary Material

Web_Material_uhaf054

## Date availability

Data are available in figures of the article and its supplementary materials.
