# Supplementary material for: Carotene hydroxylase DcCYP97A3 affects carotenoid metabolic flow and taproot color by influencing the conversion of α-carotene to lutein in carrot
Source: Hortic Res. 2025 Feb 18;12(6):uhaf054. doi: 10.1093/hr/uhaf054 (PMC12017800; doi:10.1093/hr/uhaf054)
Supplement: Web_Material_uhaf054 [file web_material_uhaf054.zip › 20250127 Supplemental Figures sub.pdf]

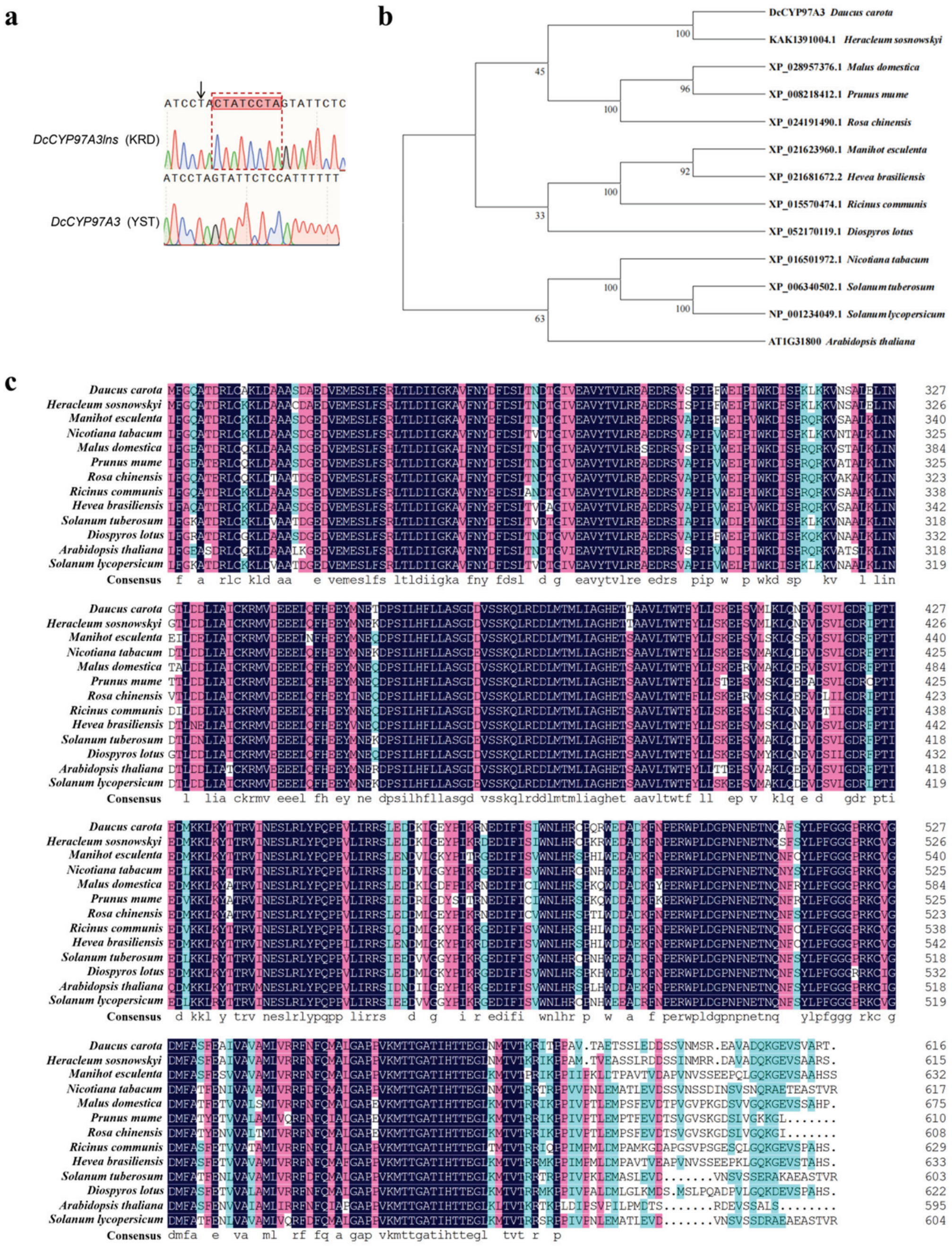

Figure S1. Sequence analysis of *DcCYP97A3* of carrot.

a: Sequencing peak map of DNA sequence fragments of *DcCYP97A3*. The dashed box indicates the inserted 8-nucleotide sequence, and the arrow indicates the position of the 1074th nucleotide of the *DcCYP97A3* exon sequence. b-c: Phylogenetic analysis (b) and multiple alignment (c) of amino acid sequences of CYP97As. KRD: orange carrot 'Kurodagosun'; YST: yellow carrot 'Yellowstone'.

```
1      ATGGCCACCACTCTCAATCTTCATCGTTTTCCAGCCCAACAATTCCTCCAATCCAAGATTCAACACAATAGAAGAAACCCCACTTCCACT
        M  A  T  T  L  N  L  H  R  F  P  A  Q  Q  F  L  Q  S  K  I  Q  H  N  R  R  N  P  T  S  T
91     TTCACCAAATTAAATGGGTCTTACAGATTTTCTGGAATCAAATGCTCTGTTTCAAATGGGAAGCTGCCTAATTCAACTGAGGAAGATGAG
        F  T  K  L  N  G  S  Y  R  F  S  G  I  K  C  S  V  S  N  G  K  L  P  N  S  T  E  E  D  E
181    GAGGAGGAGAAGAAGAAGAGGATCCGGGCTGAATTGTCTGCTCGAATTGCTTCTGGAGAATTTACAGTTGAGAAATCAGGTTTCCAGTCA
        E  E  E  K  K  K  R  I  R  A  E  L  S  A  R  I  A  S  G  E  F  T  V  E  K  S  G  F  Q  S
271    CAGTTGTTGGACGGTTTGGCAAAATTGGGGGCTCCCAGTGAGGTACTCGATGTCTTATCTAAGTGGATTGGTGCCAGTGAAGACTATCCA
        Q  L  L  D  G  L  A  K  L  G  A  P  S  E  V  L  D  V  L  S  K  W  I  G  A  S  E  D  Y  P
361    AAGATTCCAGAGGCAAAAGGAGCTATTAGTGCTATTAGAAGCGAAGCCTTTTTCATCCCTCTGTATGAGCTTTACCTCACTTACGGTGGA
        K  I  P  E  A  K  G  A  I  S  A  I  R  S  E  A  F  F  I  P  L  Y  E  L  Y  L  T  Y  G  G
451    GTTTTTAGATTGACATTTGGTCCAAAGTCCTTTTTGATAGTTTCTGATCCTACTGTAGCCAAGCACATATTAAGGGATAACTCTAAGGCT
        V  F  R  L  T  F  G  P  K  S  F  L  I  V  S  D  P  T  V  A  K  H  I  L  R  D  N  S  K  A
541    TATTCAAAAGGTATCTTGGCAGAAATCTTAGAGTTTGTAATGGGTAAAGGACTTATCCCAGCTGATGGGGAAATATGGCGTGTTAGACGA
        Y  S  K  G  I  L  A  E  I  L  E  F  V  M  G  K  G  L  I  P  A  D  G  E  I  W  R  V  R  R
631    CGTGCTATAGTCCCCGCATTGCATCAGAAATATGTAACTGCAATGATAAGCATGTTTGGACAAGCAACAGATCGCTTGTGCGCAAAGCTG
        R  A  I  V  P  A  L  H  Q  K  Y  V  T  A  M  I  S  M  F  G  Q  A  T  D  R  L  C  A  K  L
721    GATGCTGCTGCATCTGATGCAGAGGATGTGGAGATGGAGTCACTTTTTTCCCGTCTAACTTTGGATATTATTGGAAAAGCTGTCTTCAAT
        D  A  A  A  S  D  A  E  D  V  E  M  E  S  L  F  S  R  L  T  L  D  I  I  G  K  A  V  F  N
811    TATGACTTTGACTCTTTAACAAATGATACTGGAATAGTAGAGGCTGTATACACCGTCCTGCGGGAAGCGGAAGATAGAAGTGTTTCCCCA
        Y  D  F  D  S  L  T  N  D  T  G  I  V  E  A  V  Y  T  V  L  R  E  A  E  D  R  S  V  S  P
901    ATCCCATTCTGGGAAATTCCCATTTGGAAAGACATTTCGCCAAAGCTTAAAAAGGTGAATTCAGCGCTCGAGTTGATAAATGGAACACTG
        I  P  F  W  E  I  P  I  W  K  D  I  S  P  K  L  K  K  V  N  S  A  L  E  L  I  N  G  T  L
991    GATGATCTTATTGCCATATGTAAGAGAATGGTAGATGAAGAAGAGTTACAATTTCACGAAGAATACATGAATGAAACAGATCCTAGTATT
        D  D  L  I  A  I  C  K  R  M  V  D  E  E  E  L  Q  F  H  E  E  Y  M  N  E  T  D  P  S  I
1081   CTCCATTTTTTGTTGGCATCTGGAGATGACGTCTCAAGTAAGCAGCTCCGTGATGATCTGATGACAATGCTTATAGCTGGACATGAAACA
        L  H  F  L  L  A  S  G  D  D  V  S  S  K  Q  L  R  D  D  L  M  T  M  L  I  A  G  H  E  T
1171   ACTGCTGCAGTGTTGACATGGACTTTTTATCTTCTTTCCAAGGAACCTAGTGTCATGTTGAAGCTTCAAAATGAGGTTGATTCAGTTTTA
        T  A  A  V  L  T  W  T  F  Y  L  L  S  K  E  P  S  V  M  L  K  L  Q  N  E  V  D  S  V  L
1261   GGGGATAGAATTCCGACCATTGAAGACATGAAGAAACTTAAGTATACAACTCGAGTGATCAATGAATCATTGAGGCTCTACCCACAACCA
        G  D  R  I  P  T  I  E  D  M  K  K  L  K  Y  T  T  R  V  I  N  E  S  L  R  L  Y  P  Q  P
1351   CCGGTTCTGATCCGACGCTCTCTTGAAGATGACAAACTTGGCGAGTACCCAATAAAAAGGAATGAAGATATATTCATTTCAATCTGGAAC
        P  V  L  I  R  R  S  L  E  D  D  K  L  G  E  Y  P  I  K  R  N  E  D  I  F  I  S  I  W  N
1441   TTACATCGCTGTCCTCAAAGGTGGGAAGATGCAGATAAATTTAATCCTGAAAGGTGGCCTTTAGATGGACCCAACCCAAATGAGACCAAC
        L  H  R  C  P  Q  R  W  E  D  A  D  K  F  N  P  E  R  W  P  L  D  G  P  N  P  N  E  T  N
1531   CAGGCTTTTAGCTATTTACCCTTTGGTGGAGGACCAAGGAAGTGTGTAGGGGACATGTTTGCATCATTTGAGGCTATAGTAGCAGTTGCA
        Q  A  F  S  Y  L  P  F  G  G  G  P  R  K  C  V  G  D  M  F  A  S  F  E  A  I  V  A  V  A
1621   ATGCTTGTTCGTCGATTTAACTTCCAAATGGCACTTGGCGCTCCTCCTGTGAAAATGACTACAGGGGCAACCATACACACAACAGAAGGG
        M  L  V  R  R  F  N  F  Q  M  A  L  G  A  P  P  V  K  M  T  T  G  A  T  I  H  T  T  E  G
1711   TTGAATATGACAGTTACAAAAAGAATAACACCTCCAGCAGTTACAGCAGAGACATCGTCGTTGGAAGATGATTCCTCCGTAAACATGAGT
        L  N  M  T  V  T  K  R  I  T  P  P  A  V  T  A  E  T  S  S  L  E  D  D  S  S  V  N  M  S
1801   AGAGAGGCTGTAGCTGATCAGAAAGGTGAAGTTTCTGTAGCACGGACTTAG
        R  E  A  V  A  D  Q  K  G  E  V  S  V  A  R  T  *
```

Figure S2. Nucleotide acid and deduced amino acid sequence of *DcCYP97A3* gene from'YST'.

* represents the stop codon.

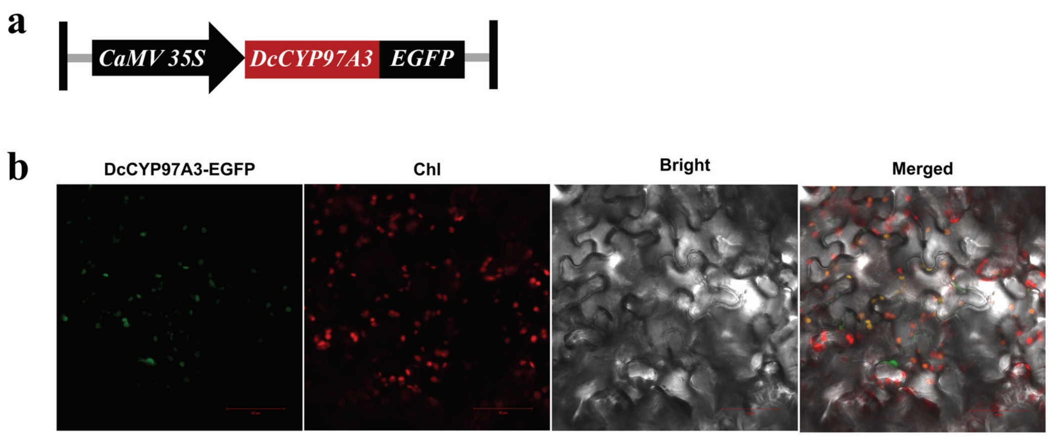

Figure S3. Subcellular localization of DcCYP97A3.

a: The schematic diagram of pSPYE-*DcCYP97A3* construct. b: Transient expression of DcCYP97A3-EGFP in tobacco leaves . Chl, Chloroplast.

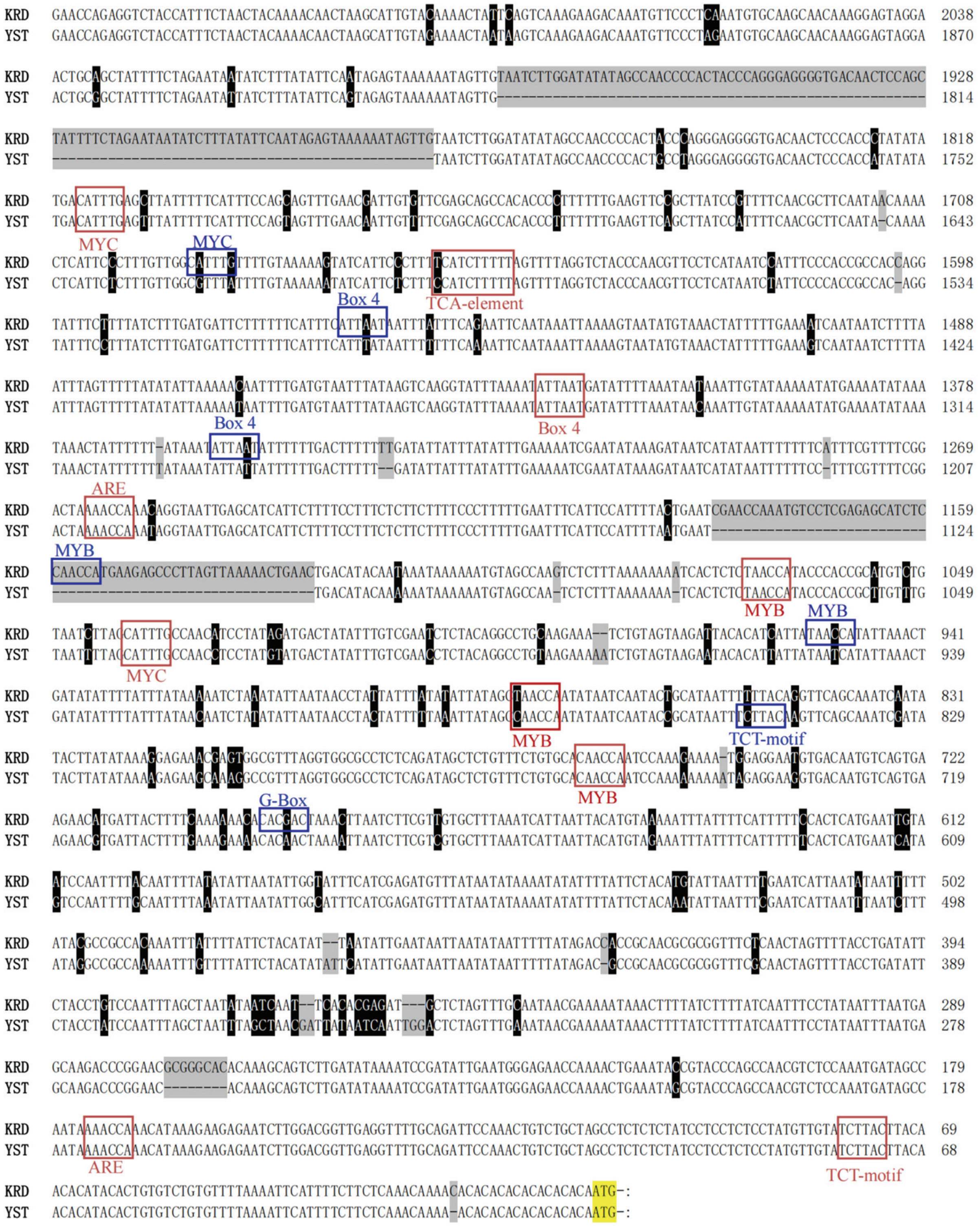

Figure S4. Sequence alignment of *DcCYP97A3* promoter region cloned from 'KRD' and 'YST'.

The gray background indicates the insertion or deletion of two promoters; Black background indicates SNPs ; The yellow background indicates the start codon ATG; In the boxs are some predicted *cis*-elements.
